# The two facets of receptor tyrosine kinase in cardiovascular calcification—can tyrosine kinase inhibitors benefit cardiovascular system?

**DOI:** 10.3389/fcvm.2022.986570

**Published:** 2022-09-27

**Authors:** Ainun Nizar Masbuchin, Widodo, Mohammad Saifur Rohman, Ping-Yen Liu

**Affiliations:** ^1^Institute of Clinical Medicine, College of Medicine, National Cheng Kung University, Tainan, Taiwan; ^2^Department of Cardiology and Vascular Medicine, Faculty of Medicine, Universitas Brawijaya, Malang, Indonesia; ^3^Department of Biology, Faculty of Mathematics and Natural Science, Universitas Brawijaya, Malang, Indonesia; ^4^Division of Cardiology, Internal Medicine, National Cheng Kung University Hospital, College of Medicine, National Cheng Kung University, Tainan, Taiwan

**Keywords:** receptor tyrosine kinase, tyrosine kinase inhibitor, vascular calcification, heart valve calcification, aortic stenosis, cardiotoxicity, cardio-oncology

## Abstract

Tyrosine kinase inhibitors (TKIs) are widely used in cancer treatment due to their effectiveness in cancer cell killing. However, an off-target of this agent limits its success. Cardiotoxicity-associated TKIs have been widely reported. Tyrosine kinase is involved in many regulatory processes in a cell, and it is involved in cancer formation. Recent evidence suggests the role of tyrosine kinase in cardiovascular calcification, specifically, the calcification of heart vessels and valves. Herein, we summarized the accumulating evidence of the crucial role of receptor tyrosine kinase (RTK) in cardiovascular calcification and provided the potential clinical implication of TKIs-related ectopic calcification. We found that RTKs, depending on the ligand and tissue, can induce or suppress cardiovascular calcification. Therefore, RTKs may have varying effects on ectopic calcification. Additionally, in the context of cardiovascular calcification, TKIs do not always relate to an unfavored outcome—they might offer benefits in some cases.

## Graphical Abstract

Summary of evidence of TK's involvement in cardiovascular calcification. The TKs can either induce or suppress calcification depend on the TK subtype, ligand, and tissue. The increase of vessel calcification can be induced by the activation of EGFR, PDGFR, FGFR, DDR, c-MET, TIE2, and ROR **(left upper panel)**, whereas the suppression on vessel calcification can be due to the activation of FGFR, IGFR, TRK, and AXL **(left lower panel)**. In the valve of heart tissue, the activation of FGFR, TRK, TIE2, and VEGFR can increase heart's valve calcification **(right upper panel)**. In contrast, the EGFR, IGFR and FGFR activation suppress calcification of heart's valve **(right lower panel)**. This dual role of TK in cardiovascular calcification might explain the various effect of TKI in cardiovascular calcification. 
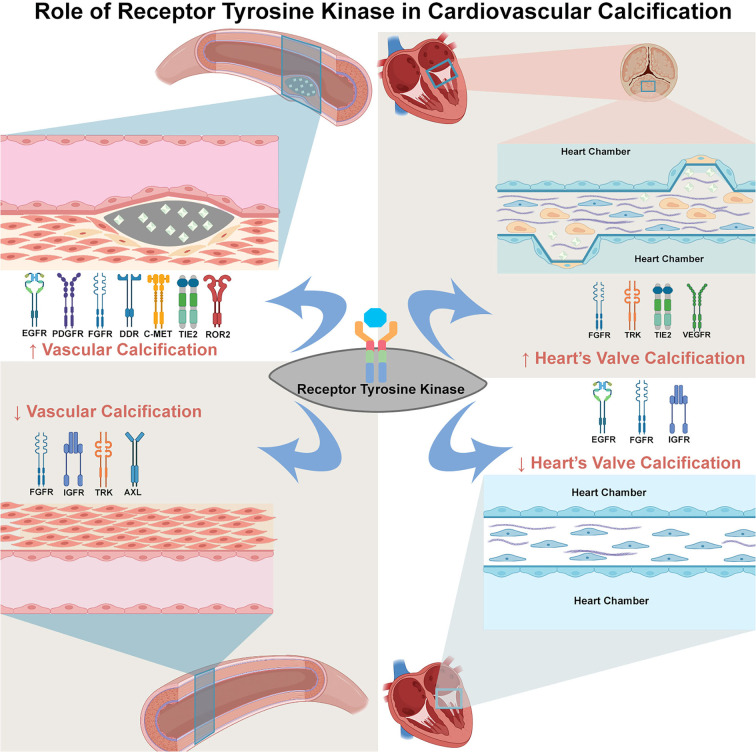

## Introduction

Tyrosine kinase (TK) is a member of the big protein kinase family, whose function is to transfer the phosphate group of adenosine triphosphate (ATP) to a specific target substrate, either protein or lipid, and it is classified into receptor tyrosine kinase (RTK) and non-receptor tyrosine kinase (NRTK) (1). It is involved in many regulatory processes, including cell proliferation and differentiation. Uncontrolled activation of TK eventually leads to cancer formation (2). Therefore, on this basis, TK inhibitors (TKIs) are widely employed in cancer treatment (3). However, TKIs have been associated with toxicity, particularly to the cardiovascular system. They have been associated with the increasing incidence of hypertension, heart failure (HF), left ventricular systolic dysfunction, myocardial ischemia (MI), and QT interval prolongation (4, 5). The cardiovascular adverse effects of TKIs are summarized in Table 1. Recent accumulated findings have demonstrated the role of TK signaling in ectopic cardiovascular calcification, which is further discussed in the next section of this review. The scope of this review was to provide a summary of evidence on RTK in cardiovascular calcification, particularly heart vessel and valve calcification.

**Table 1:** The adverse effects of TKIs on the cardiovascular system.

**Clinical**	**Number of**	**TKI drug**	**Target**	**Disease**	**Serious cardiovascular toxicity**						**Other**
**trial identifier**	**participants**				**(require hospitalization or prolong hospitalization)**						**cardiovascular toxicity**
					**ACS (angina, STEMI, NSTEMI)**	**Myocardial infarct**	**All arrythmia (AF, VT, and SVT)**	**Cardiac failure (all HF)**	**CAD**	**CVA (all stroke)**	**Hypertension**
NCT00322452	607	Gefitinib	EGFR	NSCLC	0.16%	0.16%	–	0.16%	–	0.65%	–
NCT00471497	280	Imatinib	PDGFR, c-KIT, BCR-ABL	CML	1.07%	0.71%	0.36%	0.71%	0.36%	0.36%	9.29%
NCT00471497	277	Nilotinib	PDGFR, c-KIT, BCR-ABL	CML	5.41%	3.98%	1.80%	0.72%	3.61%	5.05%	20.22%
NCT02053376	43	Regorafenib	Multikinase	Cholangiocarcinoma	–	–	–	–	–	2.33%	51.16%
NCT00076011	52	Axitinib	PDGFR, VEGFR	Kidney neoplasm	1.92%	1.92%	1.92%	–	–	1.92%	59.62%
NCT01761266	475	Sorafenib	Multikinase	HCC	–	0.42%	0.42%	0.21%	–	0.63%	30.95%
NCT01761266	476	Lenvatinib	VEGFR, FGFR, PDGFR	HCC	–	0.84%	0.21%	0.42%	0.21%	1.68%	42.02%

NSCLC, non-small cell lung cancer; CML, chronic myeloid leukemia; HCC, hepatocellular carcinoma; ACS, acute coronary syndrome; STEMI, ST elevation myocardial infarction; NSTEMI, non-ST elevation myocardial infarction; AF, atrial fibrillation; VT, ventricular tachycardia; SVT, supraventricular tachycardia; HF, heart failure; CAD, coronary artery diseases; CVA, cardiovascular accident. The clinical trial data are retrieved from the clinical trial registry (www.clinicaltrials.gov).

Vascular calcification (VC) has been identified as an independent predictor of mortality and morbidity in cardiovascular disease. It is a form of ectopic calcification, that is, mineralization outside the bone tissue. Two types of vascular calcifications, intimal and medial calcification, exist. Both types have their own risk factors. The intimal calcification commonly occurs in atherosclerosis, whereas medial calcification is frequently observed in patients with chronic kidney disease (CKD) and diabetes mellitus (6).

## A brief overview and update in the mechanism of vascular calcification

Regulation of VC is similar to bone mineralization involving the imbalance of calcification inhibitors and inducers, cell death, calcium and phosphate ion imbalance, calciprotein particles, matrix vesicles, and matrix modification (7). Calcification inducers include phosphate, bone morphogenic protein (BMP)-2, 4, 6, transforming growth factor (TGF) β, alkaline phosphatase (ALP), and fibroblast growth factor 23 (FGF23). Calcification inhibitors comprise matrix Gla protein (MGP), fetuin-A, osteoprotegerin (OPG), and inorganic pyrophosphatase (6). The role of programmed cell death (apoptosis) in VC has been reviewed elsewhere (8). In brief, apoptosis precedes calcium deposition in the matrix while calcium is accumulated within the apoptotic body. Moreover, the apoptotic cells serve as nucleating sites for calcium crystals. Matrix vesicles (MVs) are released by osteoblasts and vascular smooth muscle cells (VSMCs) as well. Healthy VSMCs secrete MVs containing calcification inhibitors. Finally, the disruption of calcium and phosphate homeostasis in certain diseases, such as CKD, can cause vascular calcification (7). Other novel drivers for VC are autophagy and mitochondrial dysfunction. Autophagy has multiple functions in VC, as reviewed elsewhere (9). It affects VC by interfering with osteogenic differentiation of VSMCs, inhibition of apoptosis, and regulation of MV release. Additionally, the treatment of VC targeting autophagy is currently in clinical trials. Mitochondrial dysfunction is currently an emerging mechanism of VC. The loss of mitochondrial function has been reported to shift the VSMC phenotype into osteoblast-like cells. Reactive oxygen species (ROS) generation induced by mitochondrial dysfunction can lead to VSMC calcification (10).

## The classification, structure, and signaling of tyrosine kinase

Protein kinase is responsible for the transfer of phosphate (phosphorylation) to targeted proteins. Phosphorylation is a post-translational modification that serves as cell signaling/communication. Thus, protein kinase is involved in many cellular processes, including proliferation and differentiation, and, potentially, diseases. Protein kinase comprises eight members, which are TK, tyrosine kinase-like (TKL) kinase, serine/threonine kinase (STE), casein kinase (CK1), protein kinase A, G, C (AGC), calcium/calmodulin-dependent protein kinase (CAMK), CDK-MAPK-GSK-CDK-like kinases (CMGC), and receptor guanylate cyclase (RGC). Among them, TK is the most widely studied and has the highest expression and the most extensive distribution among human kinases (11).

**Figure 1:**
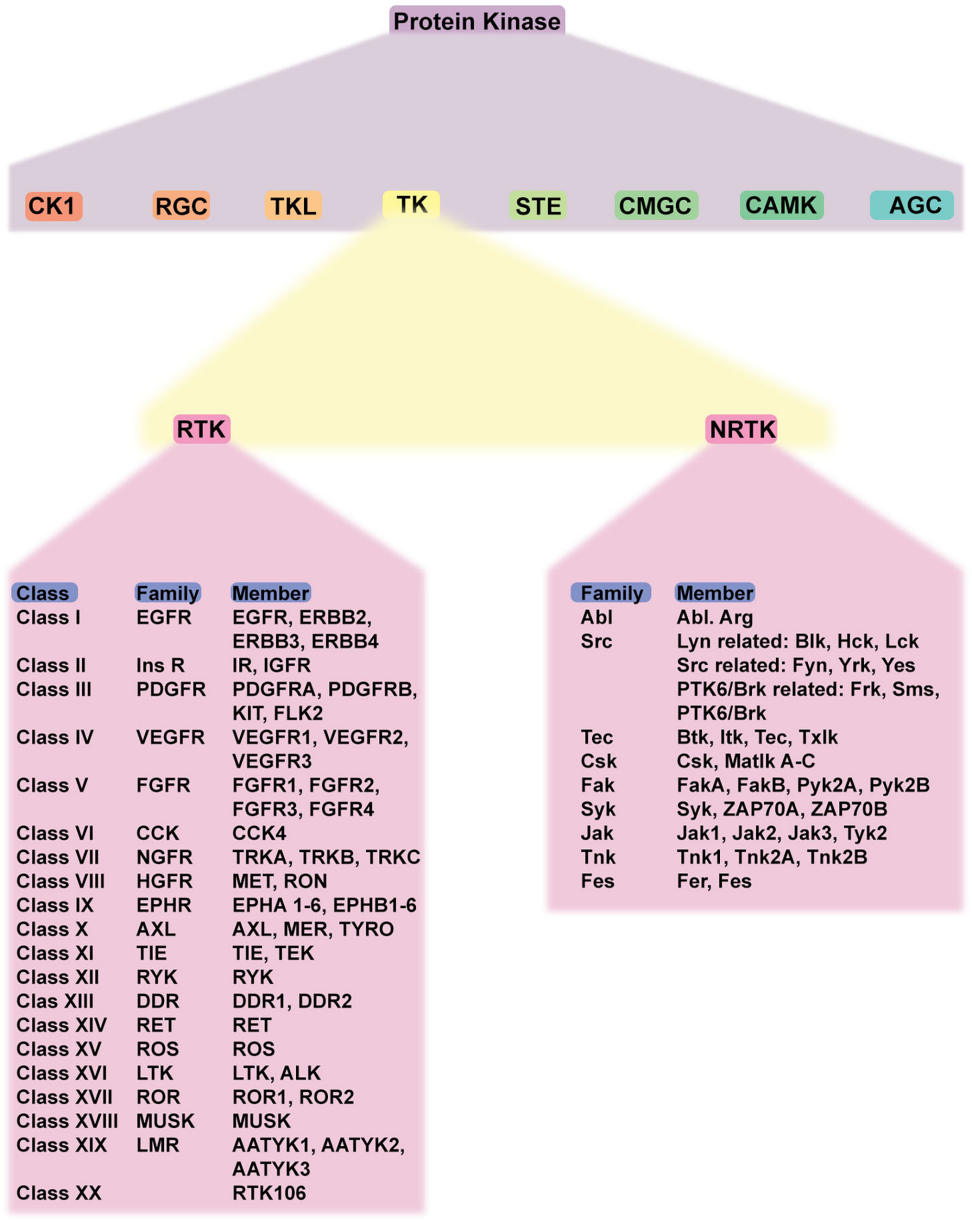
Schematic classification of protein kinases and detailed classification of tyrosine kinases. Protein kinase has eight members, including TK. TK is classified into RTK and NRTK which are subdivided into classes and families. Abbreviations: CK1, casein kinase; RGC, receptor guanylate cyclase; TKL, tyrosine-kinase like; TK, tyrosine kinase; STE, serine/threonine kinase; CMGC, CDK-MAPK-GSK-CDK-like kinase; AGC, protein kinase A, G, C; RTK, receptor tyrosine kinase; NRTK, non-receptor tyrosine kinase; EGFR, epidermal growth factor receptor; PDGFR, platelet-derived growth factor receptor; VEGFR, vascular endothelial growth factor receptor; FGFR, fibroblast growth factor receptor; CCK, cholecystokinin; NFGR, nerve growth factor receptor; HGFR, hepatocyte growth factor; EPHR, ephrin receptor; AXL, anexelekto receptor; TIE, Tek receptor tyrosine kinase; ALK, anaplastic lymphoma kinase; RYK, receptor-like tyrosine kinase; ROR, receptor tyrosine kinase-like orphan receptors; MUSK, muscle-specific kinase.

As a member of protein kinases, TK also plays a crucial role. It is involved in the regulation of cell growth, differentiation, adhesion, motility, and death. TK is classified as RTK and NRTK. RTK comprises 20 classes/families: EGFR family, insulin receptor family, PDGFR family, VEGFR family, FGFR family, CCK receptor family, NGFR family, HGFR family, Eph receptor family, AXL receptor family, TIE receptor family, RYK receptor family, DDR family, RET receptor family, ROS receptor family, LTK receptor family, ROR family, MusK receptor family, and LMR receptor family. NRTK comprises 10 families: ABL family, ACK family, CSK family, FAK family, FES family, FRK family, JAK family, SRC family, TEC family, and SYK family. Considering the structure, RTK consists of extracellular, transmembrane, and cytoplasmic domains. In the absence of a ligand, RTK is monomeric (except for Met and its family and insulin receptor family). The cytoplasmic domain contains tyrosine kinase catalytic domains and a non-catalytic carboxyl-terminal region. Most autophosphorylation occurs in the non-catalytic region (12). NRTKs, in contrast, lack extracellular ligand-binding domain and are located within the cytoplasm, while some are anchored to the cell membrane. NRTKs have a special domain mediating protein–protein, protein–lipid, and protein–DNA interactions. SH2 and SH3 are common domains found in NRTKs (13). The classification of TKs is summarized in Figure 1.

**Figure 2:**
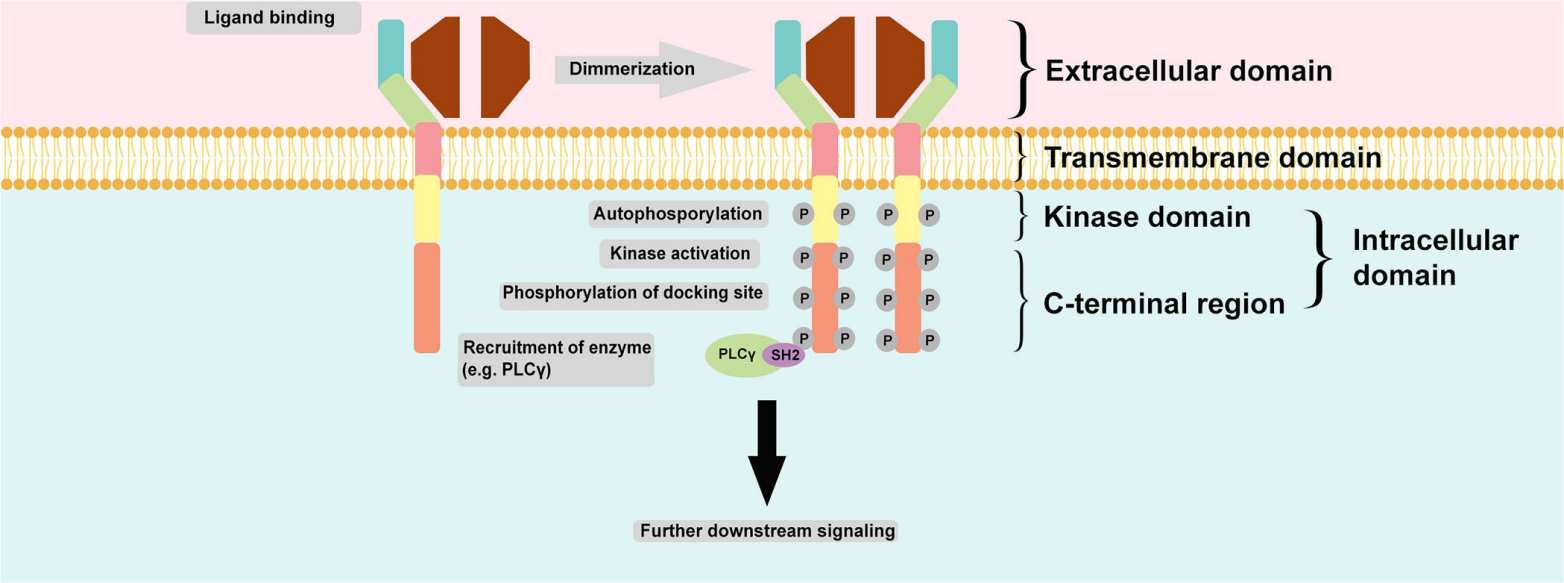
Common structure of RTK and its mechanism of activation and signaling. RTK is structurally composed of an extracellular domain which interacts with its ligand and distinguishes it from NRTK; an anchoring transmembrane domain; and an intracellular domain which composed of kinase domain and C-terminal region. Commonly, two monomeric RTKs are dimerized upon ligand binding leading autophosphorylation and subsequent tyrosine kinase activity stimulation and finally phosphorylation of docking site within the C-terminal region of intracellular domain for docking with another enzyme, such as PLCγ. The final action is further downstream signaling activation.

Regulation of RTK activation requires two processes: enhancement of the intrinsic catalytic activity of the protein and creation of a binding site to recruit downstream signaling proteins. These two processes can be activated mainly through tyrosine autophosphorylation. Autophosphorylation of the kinase domain stimulates kinase activity, whereas autophosphorylation of tyrosine on the carboxyl-terminal region generates a docking site for the modular domain recognizing phosphotyrosine. The most common phosphotyrosine-binding (PTB) molecules are the SH2 domain and PTB domain. Autophosphorylation is induced upon receptor oligomerization induced by a ligand. However, for receptor binding to a dimeric ligand, a receptor dimer is likely to form. Thus, RTK dimerization could also induce autophosphorylation (13). Figure 2 summarizes the common structure of RTK and its mechanism of activation and signaling.

## Role of receptor tyrosine kinase in cardiovascular diseases

Given the wide and important function of TK in many organs, it can be involved in many diseases. Tyrosine kinase has been associated with cancer. Many cancers have been associated with mutations of TKs. For example, epidermal growth factor receptor (EGFR) mutation has been reported as a driver of breast cancer. Interestingly, TK dysfunction has been reported to affect the physiology of the heart and vessels. Therefore, RTK signaling disruption could lead to cardiovascular diseases. This concept is proven by the increased cardiovascular toxicity of TKIs in cancer patients (14). Other reported TKIs-related cardiotoxicity includes hypertension and abdominal aortic aneurysm development (15). The summary of TKI-related cardiovascular adverse effects based on clinical trial data is presented in Table 1. Here, we also briefly discuss selected cardiovascular diseases caused by RTK dysregulation. This review emphasizes the role of RTK in cardiovascular calcification.

### Atherosclerosis

EGFR is expressed in the cells involved in atherosclerosis including vascular and immune cells. A recent study explored the function of EGFR in the development of atherosclerosis (16, 17). The specific deletion of *EGFR* in myeloid cells resulted in the reduction of macrophage and necrotic size. The scavenger receptor for lipid uptake (CD36) was also reduced in the *EGFR*-knocked out macrophage. Additionally, the specific EGFR inhibition in CD4+ limits atherosclerotic plaque by inducing CD4+ anergy.

### Hypertension

Several RTKs (VEGFR, PDGFR, and EGFR) are involved in the regulation of vascular tone. VEGF through its receptor, VEGFR, has been reported to promote nitric oxide (NO) production which is well known for its vasodilating effect (18). Several RTKs can crosstalk with G-protein coupled receptor (GPCR) leading to transactivation of RTK through GPCR, such as angiotensin II type 1 receptor (AT1R) which is the receptor for angiotensin II. The transactivation of PDGFR and EGFR through AT1R could be the basis of these two receptors in regulating blood pressure (19). Furthermore, angiotensin II, a principal mediator for regulating vascular tone, can directly activate EGFR, and the blocking of EGFR with EGFR antibody results in a decrease in blood pressure (20).

### Arrythmia

Recently, a study revealed the association between EGFR ligand and the incidence of atrial fibrillation (21). The evidence of EGFR activation-induced arrythmia is provided by a molecular study reporting the activation of EGFR in reperfusion arrythmia-induced rats (22). EGFR phosphorylation increased in arrythmia. The phosphorylation of Na^+^ ion channel and Ca^2+^ ion channel increased corresponding with the increase of phosphorylation of EGFR. Interestingly, both pharmacological inhibition of EGFR and *EGFR* knockdown reduced the incidence of ventricular fibrillation induced by reperfusion.

### Heart failure

Cardiomyocytes have been reported to express RTK. The specific *EGFR* overexpression in the cardiomyocytes resulted in the induction of its pro-survival pathway (23), suggesting its role in cardiomyocyte function. EGFR has been documented to be involved in the preservation of cardiac function and tissue homeostasis (24). The evidence of heart failure following Osimertinib (EGFR inhibitor) use and improvement following its cessation has been reported (25).

## Role of receptor tyrosine kinase in cardiovascular calcification

### Epidermal growth factor receptor

EGFR is the most widely studied RTK, and its mechanism has been intensively investigated in cancer. The EGFR family consists of four related tyrosine kinase receptors: EGFR (ErbB1), ErbB2, ErbB3, and ErbB4. Upon EGFR binding with its ligand, EGFR becomes dimerized and leads to autophosphorylation of its tyrosine residue and activates their downstream signaling targets, including MAPK, PI3K, PLCγ, and Src-kinase, leading to cell proliferation, survival, differentiation, migration, and matrix regulation. Additionally, in the absence of a ligand, EGFR can be activated through a mechanism called transactivation *via* the crosstalk with others, for example, G protein-coupled receptor activation (24, 26). EGFR is expressed in VSMCs and cardiomyocytes. Neither the impairment of embryonic heart development nor placental function is affected by the global knockout of *Egfr* (24).

**Figure 3:**
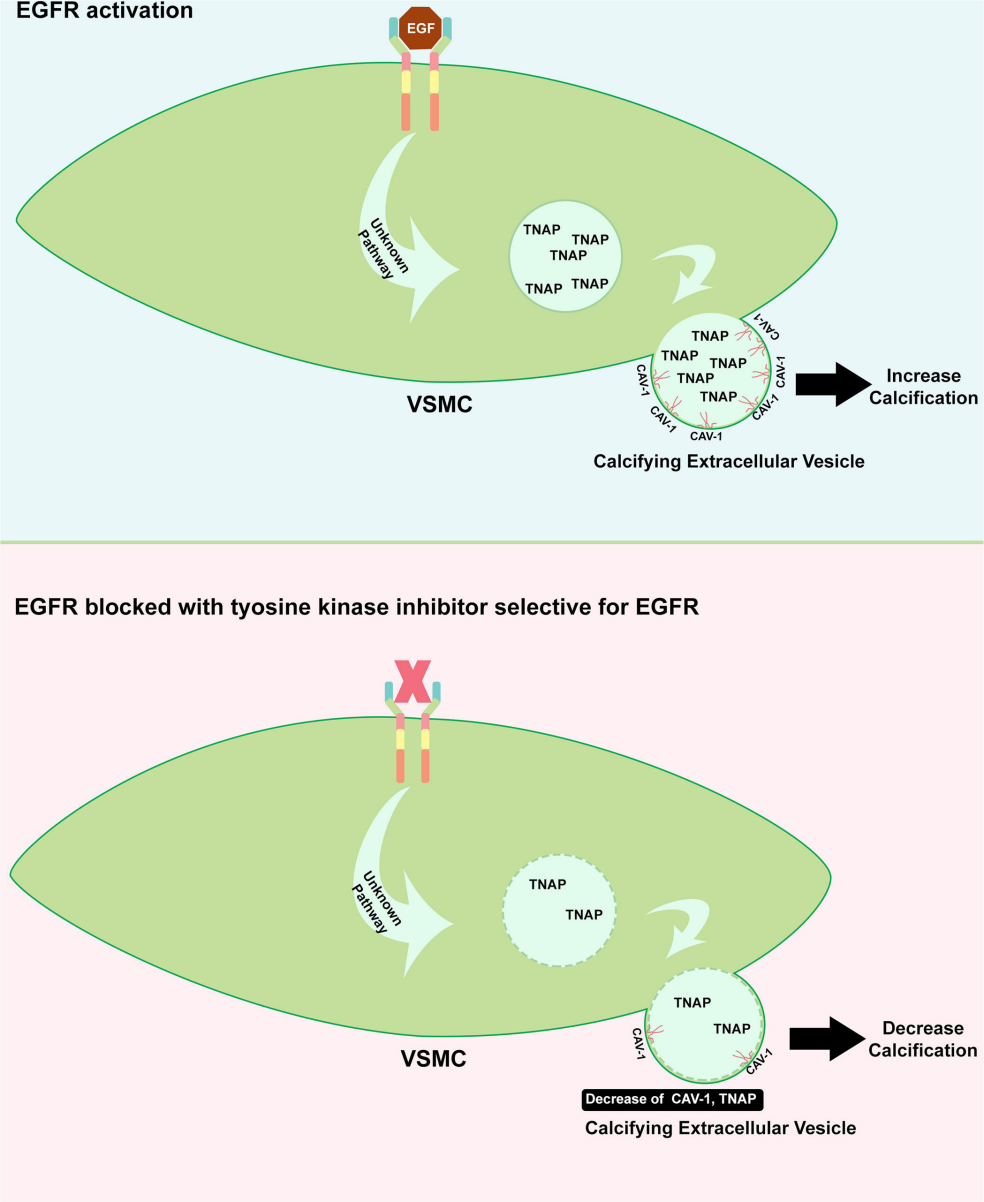
Role of EGFR in inducing calcification. Upon EGFR activation on VSMC, the formation of CAV-1 positive vesicle is increased including the augmentation of TNAP which can inhibit the calcification inhibitor. The release of this enzyme to extracellular matrix could cause imbalance of inhibitor and inducer of calcification **(upper panel)**. TKI which specifically target EGFR could benefit the cardiovascular system where it protects the vessel from developing calcification, in part, through the decrease of CAV-1 positive vesicle containing TNAP **(bottom panel)**.

The evidence of EGFR involvement in the development of VC arises from the study of Nik et al. (27), as illustrated in Figure 3. In a genetic association study, *EGFR* mutation has been strongly associated with the incidence of VC. These data have been further supported by the clinical evidence that serum EGFR was significantly higher in subjects with VC. In an animal study using CKD to induce calcification and *in vitro* study using human VSMCs, EGFR blockage using a TKI, AG1478, resulted in a decrease in VC. A further molecular study has shown that EGFR inhibition prevented the formation of CAV1-positive calcifying extracellular vesicles. Calcifying extracellular vesicle is known to cause ectopic calcification within the vessel. Moreover, the activity of tissue non-specific alkaline phosphatase (TNAP), which is involved in the inactivation of a common calcification inhibitor, inorganic pyrophosphate, has been reduced with the inhibition of EGFR. The EGFR blockage has not induced VSMC phenotype changes, suggesting that VC reducing effect is mediated independently of phenotype switching, which is a common mechanism of VC. According to this, EGFR signaling has been reported to play a critical role during endochondral ossification (28). Additionally, crosstalk between EGF and BMP9 signaling which is associated with osteogenic differentiation has been evidenced (29). Interestingly, this crosstalk can be blunted with erlotinib and gefitinib (TKIs that specifically inhibit EGFR) suggesting and additional link with calcification.

Recent findings by Barrick et al. have shown that EGFR perturbance leads to the development of calcific aortic stenosis (30). *Egfr* global knockout resulted in the increase of osteoblast cells in the aortic valve. Recently, Wu's group has revealed that specific *Egfr* deletion resulted in calcified aortic stenosis. The valvular interstitial cells (VICs) showed an increase in their proliferation and dedifferentiation into cells secreting and depositing extracellular matrix and bone-like formation and calcification. The expression of genes involved in epithelial-to-mesenchymal cell and osteogenic activity has been increased (31). These findings suggest that EGFR signaling is required to protect the heart valve from calcification.

However, in another study, EGFR inhibition has been reported to improve atherosclerosis (17). EGFR is expressed in CD4 T cells. Inhibition of EGFR using erlotinib reduced T cell proliferation, activation, and migration within an atherosclerosis lesion in *Ldlr-/-* mice and protected against atherosclerotic progression and development. Specific *Egfr* knockout in CD4 T cells impacted the decrease in T cell proliferation and activation both *in vivo* and *in vitro*, as well as the reduction of interferon (IFN)γ, interleukin (IL)-4, and IL-2 production. EGFR engagement is indeed important for macrophage proatherogenic activity.

VSMCs have been reported to express EGFR. *EGFR-specific* knockout has reduced VSMC proliferation (24). The blockage of EGFR using monoclonal antibodies has resulted in the reduction of intimal hyperplasia and increment in re-endothelialization in the balloon-injury rat. EGFR has an important role in VSMC proliferation and migration (32), as its inhibition mitigates proliferation and migration induced by balloon injury (33). A recent study has reported that TKI, gefitinib, can specifically inhibit VSMC proliferation by inhibiting EGFR-Akt phosphorylation (34).

### Platelet-derived growth factor receptor (PDGFR)

PDGFR is a transmembrane tyrosine kinase, which exists in two types, PDGFR-α and PDGFR-β. Those two receptors control the growth of connective tissue. PDGFR-α is mainly expressed in mesenchymal cells, whereas PDGFR-β is mainly expressed in VSMCs and pericytes. The ligands of PDGFR are PDFGF-A, PDGF-B, PDGF-C, PDGF-D, homo and heterodimer PDGFAA, PDGFAB, PDGF-BB, PDGF-CC, PDGF-CC, and PDGF-DD. PDGF acts in a paracrine and autocrine manner. PDGF-B is produced by endothelial cells, megakaryocytes, and VSMCs (35). Upon PGDFR binding with its ligand, receptor conformation and receptor dimerization (either homodimerization or heterodimerization) occur, phosphorylating and activating a TK domain and subsequent downstream signaling. PDGFR-α can bind PDGF-A, -B, and -C; hence, it can also be activated by PDGF-AA, PDGF-BB, and PDGF-CC. PGFR-β binds PDGF-B and -D with high affinity; hence, it can be activated by PDGF-BB and PDGF-DD as well. PDGFR-β is less easily activated by PDGFs compared to PDGFR-α due to a large aromatic residue in the ligand-binding surface. Consequently, it is more specific and selective. PDGF-B can bind to both receptors due to the abundant presence of long-chain hydrophilic residues (36).

VC can be mediated by VSMC phenotype switching (6). Activation of PDGFR has been involved in VSMC phenotype switching through common transcription factors involved in osteoblast-like phenotype switching, such as Krüppel-like factor (KLF4) (37). Overexpression of PDGF-B in mice has led to the upregulation of PDGFR, thereby increasing VC through augmentation of Runx2 and ALP (38). One of the possible mechanisms of PDGFR activation-induced VC is through mitogen-activated protein kinase (MAPK) activation. Upon the binding of PDGFR to its ligand, the downstream signaling activates p38MAPK in VSMC (35), which, in turn, upregulates Runx2 to induce osteoblastic transdifferentiation and subsequently VC formation (39). In fact, in the bone tissue, the bone explant cultured with PDGFR inhibitor tyrosine kinase (AG1295) or PI3K inhibitor has resulted in more matrix mineralization (40), suggesting that different tissues yield different responses.

Recently, PDGF and PDGFRβ loss of function mutation have been identified to cause idiopathic basal ganglia calcification (IBGC), which is characterized by ectopic calcification in small arteries and arterioles in the brain (41). Mutation causing loss of function of PDGFRβ has been proven to disrupt the downstream signaling (42), therefore, raising a concern about the long-term use of drugs inhibiting PDGFR, such as imatinib. Imatinib is the first generation of TKIs used to treat chronic myelogenous leukemia (CML). However, the resistance issue drives scientists to develop a newer generation of drugs, such as nilotinib. The long-term use of nilotinib (a TKI) in CML patients has been reported to induce aortic stenosis after 12-months therapy initiation (43). Carracedo et al. have isolated human VICs from human aortic valves and exposed them to nilotinib and imatinib. Nilotinib promoted VIC calcification by increasing BMP2 and its subsequent signaling pathway, SMAD 1/5/8. Imatinib, however, did not lead to significant calcification nor BMP2 increase. In contrast, the expression of PDGFRα in VICs was conversely related to calcification identified in the heart valve (44).

### Fibroblast growth factor receptor (FGFR)

Recent accumulating evidence has indicated the role of FGFR in VC formation. FGFR is an RTK that has a broad function mainly related to organ development, metabolism, and disease. It also has an important role in tissue repair, regeneration, and inflammation. Similar to other tyrosine kinase receptors, FGFR comprises extracellular, transmembrane, and intercellular domains. The extracellular domain of FGFR possesses a unique immunoglobulin-like domain, which is a binding motif for FGF. FGF binding to monomeric FGFR induces conformational changes and subsequently dimerization, autophosphorylation, and finally, kinase activation (45). FGFR ligands associated with cardiovascular calcification are FGF2, FGF21, and FGF23. Current evidence, through different ligand binding, has shown the role of FGFR in suppressing (46–48) or inducing (49, 50) VC.

FGF2 binds to FGFR and regulates VSMC mineralization (46). FGF2 acts in a paracrine manner due to the lack of heparin-binding domain in its C-terminus required for the circulation and action on distant target organs (51). Borland et al. have demonstrated that FGF2 treatment abolished phosphate-induced VSMC calcification confirming its role in VC suppression. Additionally, the blockage of FGFR increased VSMC calcification through the inhibition of pAkt, pERK1/2, and ERK1/2. Intriguingly, FGFR could crosstalk with TGFβR through PKCα activation, which, in turn, inhibits TGFRβ signaling and subsequent VC (46). In concordance with vessel calcification, Lam et al. have demonstrated that FGF2-FGFR1 binding also inhibited the phenotype switching of VICs in the heart valve; hence, it maintained the quiescent, that is, normal, phenotype (52). In the calcified valve, FGF2 expression is low while FGFR1 expression is high, suggesting a possible correlation between FGFR signaling and calcific disease.

**Figure 4:**
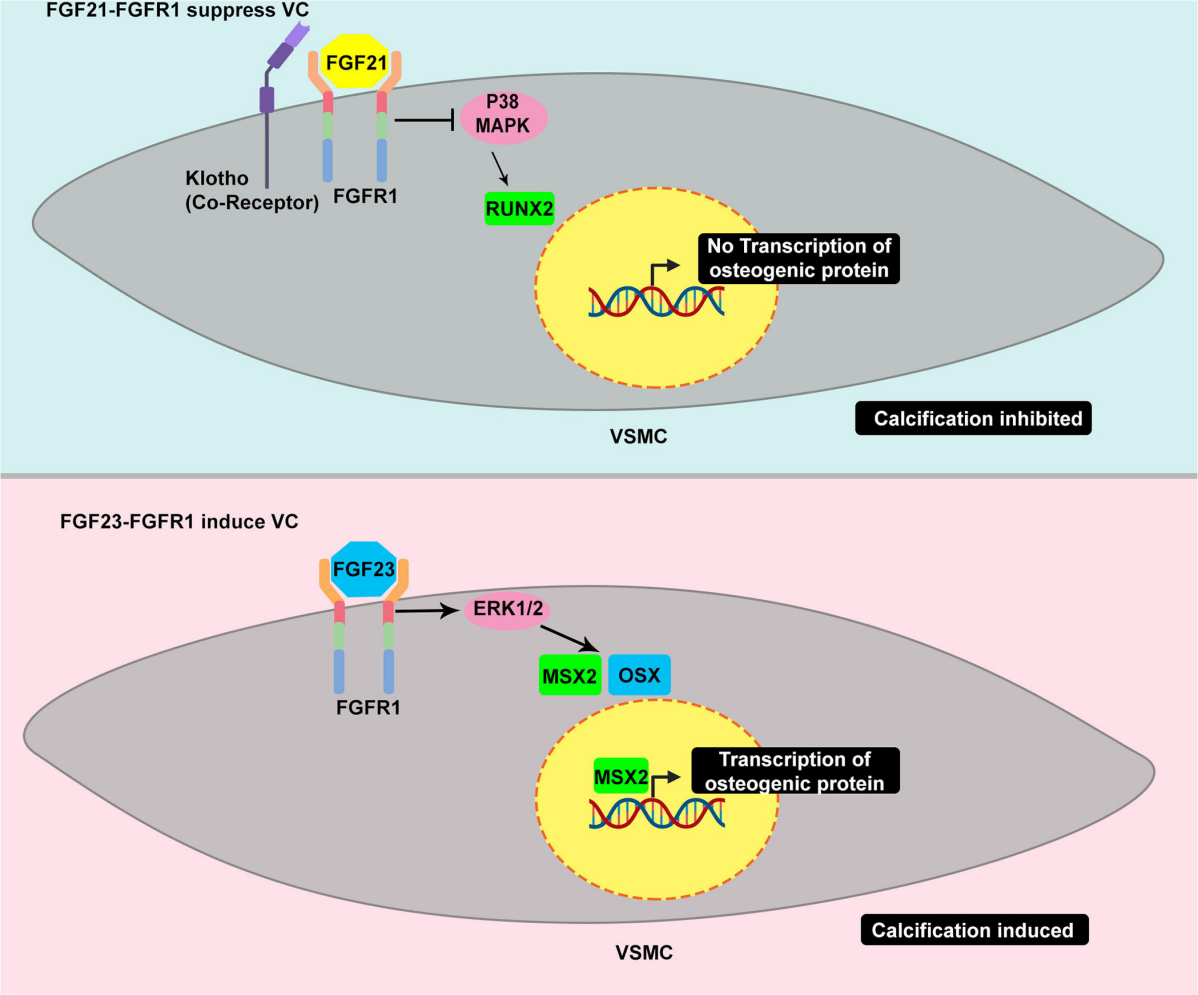
Example of TRK induced by different ligand yields different response in VC – the dual roles of TRK in vascular calcification. FGFR1 activated by FGF21 suppresses the calcification, in part, through the inhibition of p38 MAPK. The p38 MAPK induces the increase of RUNX2 which is considered as master regulator of osteoblast-like differentiation. The decrease of RUNX2 eventually affects the VSMC to osteoblastic-like cell reprogramming leading to calcification suppression **(upper panel)**. In contrast, FGFR1 activated by FGF23 results in different responses. The FGF23-FGFR1 coupling stimulates ERK1/2 which is common activated pathway in promoting calcification. This pathway leads to increase of MSX and OSX which promote osteogenic differentiation **(bottom panel)**.

It is controversial whether FGF21 is protective against or induces cardiovascular diseases. FGF21 has been strongly related to traditional cardiovascular risk factors (53). It has been clinically associated with VC in hemodialyzed patients. Serum FGF21 has been highly correlated with high calcium scores in three different thoracic aorta segments: ascending thoracic aorta, aortic arch, and descending aorta (54). Additionally, FGF21 has been correlated with the increasing grade of aortic stenosis (55). However, emerging evidence of the beneficial metabolic effect of FGF21 has been demonstrated *in vitro* and *in vivo*, indicating that it is not just a simple biomarker (53). It is tempting to speculate that the increase in FGF21 in such conditions is probably a physiological protective response. Molecularly, FGF21 has been reported to regulate vessel calcification (47). Cao et al. have demonstrated the increase in FGFRs, FGFR1 and FGFR4, transcription and a decrease in FGFR2 and FGFR3 transcription in VSMCs after phosphate induction. Treatment with FGF21 led to a decrease in calcifying markers, BMP2 and RUNX; hence, mineral deposition. Intriguingly, FGF21 treatment augmented FGFR1 and FGFR3 transcripts. Additionally, FGF21 suppressed the phosphorylation of P38. Therefore, FGFR1/P38MAPK/RUNX2 serves as a signaling basis for VC suppression by FGF21 (Figure 4). FGF21–FGFR engagement has also been reported to decrease VC through ameliorating endoplasmic reticulum stress-induced apoptosis (48) and modulating the BMP2/SMAD pathway (56).

FGF21 and FGF23 are members of the FGF19 group family possessing a unique structure, the heparin-binding domain in the C-terminus, which allows them to circulate and act in distant organs (51). Therefore, FGF21 and FGF23 are endocrine FGFs. FGF23 is a bone-derived hormone acting to decrease the level of phosphate serum. Its post-translational processes include glycosylation by a specialized enzyme, GALNT3, to prevent it from degradation and, thus, confer to the increase in FGF23 level. FGF23 has been clinically and experimentally associated with VC. FGF23 can bind to FGFR with a co-receptor called klotho, which enhances its binding affinity. FGF23–FGFR-klotho binding has been reported to induce VC. However, a study has reported that FGF23 alone is sufficient to stimulate mineralization in VSMCs through ERK1/2 stimulation and subsequent increase of MSX and OSX (Figure 4) (50). A recent study has revealed that extracellular inorganic phosphate (Pi) was capable of activating unliganded FGFR in osteocytes, thereby inducing GALNT3 production, which, in turn, glycosylates FGF23 (57). FGF23 appears to have a similar fibrocalcifying effect in VIC through FGFR1 and FGFR4. Additionally, *FGF23* knockdown and neutralization of FGF23 using soluble klotho have suppressed fibrocalcification (58).

### Insulin growth factor receptor (IGFR)

Insulin-like growth factor (IGF) system comprises two ligands (IGF1 and IGF2), six IGF-binding proteins (IGFBPs), and two receptors (IGFR1 and IGFR2). This system is crucial for cell growth, proliferation, and differentiation (59). IGFBPs transport IGF1 and IGF2 in plasma to protect them from degradation, hence, increasing their bioavailability in circulation. IGFBPs also affect IGFR signaling (60). IGF1 has 57% structural sequence similarity and 85% TK domain identity with insulin receptor (IR). However, IGFR2 is structurally distinct and lacks intrinsic TK activity (59). IGFR1 is synthesized and post-translationally modified with the involvement of N-glycosylation (6). Since it is involved in malignancy, IGFR1 has been the target of cancer drugs for years despite its failure in clinical trials (61). IGFR1 has an α subdomain-containing conserved regions involved in ligand binding and a β subdomain, in which the intracellular subunit contains TK enzymatic domain responsible for autophosphorylation. The α and β subunits are connected by a disulfide bridge (62). Evidence of IGFR involvement in VC is currently emerging (6).

In 2011, two independent groups reported that IGFR regulates VC via different mechanisms (63, 64). OPG has long been known to inhibit VC by acting as a soluble receptor for RANKL, possessing a procalcific effect (65). di Bartolo et al. have demonstrated that the IGF receptor for IGF1 is involved in VC suppression. They have revealed that high calcium caused inhibition of IGFR1 expression, thereby stimulating VC, whereas moderate calcium yielded opposite effects by inducing IGFR1 expression mediated by osteoprotegerin (OPG), hence, VC inhibition (63). Additionally, the regulation of VC is also achieved by post-translational regulation through N-glycosylation of IGFR1. Siddals et al. have shown the importance of glycosylation in VC by treating VSMCs with simvastatin, which can act as an N-glycosylation inhibitor. As expected, statin administration induced calcification by downregulating IGFR1 expression, which, in turn, decreases IGFR1 protection effects toward VC. Glycosylation of receptor or protein was required to avoid degradation (64). IGFR1 expression has been downregulated by advanced glycation products (AGEs), which are strong VC inducers, thereby stimulating VC by augmenting nuclear factor kappa B (NF-κB) expression, which, in turn, binds IGFR1 promoter at −230 to −219 position (66).

IGFR1 activation by different ligands apparently yields distinct effects in VC. IGFR1 activated by IGF1 enhanced VC by activating both ERK and PI3K pathways (67). In contrast, while IGF2, IGFR1, and IGFR2 were upregulated in calcified VSMCs, the treatment with IGF2 did not increase VC, albeit signaling showed the stimulation of Akt and ERK1/2, which is involved in VC (68). Intriguingly, IGFR1 is abundantly expressed in VIC (69). Likewise, IGF1 treatment, through IGFR1, decreases aortic valve calcification by augmenting the osteogenic differentiation of VIC. Interestingly, a dipeptidyl peptidase-4 (DPP-4) inhibitor could ameliorate aortic valve calcification by preventing IGF1 degradation, rendering it potentially a new agent for valve calcification disease (70, 71).

### Vascular endothelial growth factor receptor (VEGFR)

A recent study has revealed the abundant expression of VEGFR2 in VIC isolated from the aortic valve of patients with aortic valve stenosis (72). More recently, Li et al. have demonstrated that VICs treated with VEGF exhibit abundant Runx2—the master regulator of osteoblast-like cell differentiation. Additionally, VEGF-treated VICs had increased calcium entry and apoptotic cell numbers. The increase in calcium could be obtained through PLCγ, which is the down-signaling target of the TK receptor. PLCγ subsequently phosphorylates CaMKII to activate transcription factor CREB (73). Ectopic calcification is considered to mimic bone ossification. Recent evidence has also shown the role of VEGF in bone growth, as *VEGF*^120/120^ KO mice display reduced VEGFR expression in the perichondrium and exhibit a decrease in osteoblast activity (74).

### Axl

Axl, an RTK, belongs to the TAM family and has a wide range of functions, being involved in cancer and other diseases. Its identified ligands are Gas6, protein S, Tubby, and Galectin. Similar to other RTKs, Gas6-Axl coupling leads to its oligomerization and subsequent autophosphorylation to activate the kinase and eventually activate the downstream signal cascade (75).

The first evidence of Axl-mediated VC suppression has come from Collet et al. (76). They have demonstrated that Axl was expressed and phosphorylated in normal VSMCs and became downregulated as VSMCs were calcified. Axl overexpression attenuated VSMC calcification by activating the PI3K/Akt axis. They have further proved that treatment with a PI3K inhibitor abrogated the VC inhibition by Axl overexpression. Badi et al. have revealed that Axl downregulation by miR34a led to VSMC transdifferentiation and subsequently VC (77). Axl downregulation by inorganic phosphate has also been reported to induce VC. Axl downregulation led to a decrease in Akt phosphorylation, which, in turn, lowered VSMC survival and increased apoptosis, one of the VC mechanisms (78, 79). Recently, agents delaying VC progression have been reported. For instance, vitamin K2-induced VC inhibition can be explained through the Gas/Axl interaction, which leads to the anti-apoptotic pathway (80). Statin has been reported to suppress VC through the Gas6-Axl pathway as well (81).

VSMCs are the main players in medial calcification. Other residing cells, such as pericytes, have also been reported to orchestrate VC (82). Axl can also inhibit pericytes from being differentiated into osteogenic cells (83). Nevertheless, Hyde et al. (84) have reported that VC did not occur in nephrectomy mice fed with a high phosphate diet with *Axl-/-* background. Instead, *Axl*-deficient mice exhibited tremendous tubular-interstitial apoptosis. Finally, using Gas6-deficient mice, Kaesler et al. have not been able to prove the prominent role of Gas6 in VC (85). Collectively, the evidence indicates the role of Axl in suppressing VC.

### TRK

Tropomyosin receptor kinase (Trk) receptor family comprises TrkA, TrkB, and TrkC. These receptors are predominantly expressed in neuronal tissue. Their natural ligand is neurotrophin. Trk is encoded by *NRTK*, whose mutation can lead to Trk with an abnormal domain that constitutively activates tyrosine kinase; therefore, it is identified as an oncogene (86). Recently, Trk has been reported to be expressed in other cells, such as cardiovascular cells, and the disease caused by its abnormal signaling has been identified as well.

Jiang and Tuan (87) have recently identified the expression of TrkA in human chondrocytes, and its activation using NGF, a high-affinity ligand for TrkA, led to the enhancement of human chondrocyte matrix mineralization by upregulating Runx2 and ALP. Interestingly, pharmacological inhibition of Trk tyrosine kinase has resulted in the reduction in the marker of osteoblast activity, ALP (88). Trk has also been reported to play a role in aortic valve disease. It is involved in heart valve calcification, as evidenced by Yao et al. They have demonstrated that the activation of Trk by NT3 yielded an osteogenic response in human aortic VIC and have found that the markers of osteogenic properties, Runx2, TGFβ1, and BMP2, increased (89). Finally, the most recent evidence has reported that TrkB was expressed in endothelial cells and played a role in VC by enhancing the EndMT process. In human umbilical vein endothelial cells (HUVECs), the activation of TrkB by its ligand (BDNF; Brain-Derived Neurotrophic Factor) led to an increase in KLF2, which, in turn, decreased EndMT transdifferentiation, leading to VC decrease (90).

### Discoidin domain receptor (DDR)

DDR is an RTK that can recognize collagen and its cognate ligands. Their unique structural characteristics, that is, the presence of discoidin domain and activation kinetics distinguish them from other members of the kinase superfamily. They physiologically regulate the differentiation of cells. DDR family has two members: DDR1 and DDR2. DDRs follow a canonical pathway of tyrosine kinase signaling, in which, upon binding with their ligand, collagen, they undergo autophosphorylation and subsequent kinase activation, leading to a further downstream signaling cascade, and eventually eliciting a certain cellular program (91). DDR1 has been reported to have a crucial role in VC formation. *Ddr1* knockout in mice and VSMCs has led to a decrease in plaque and VSMC calcification, respectively (92, 93). Moreover, DDR1 induced VC through the PI3K/Akt/Runx2 pathway in a diabetic setting (92). Interestingly, Ngai et al. have demonstrated that DDR1 could sense matrix stiffness through RhoA to promote VC (94). VC pathogenesis involves the release of MVs from transdifferentiated calcifying VSMCs into the extracellular matrix. Evidence that DDR1 plays this role has been elucidated by Krohn et al. (95). Mechanistically, DDR1 directly interacts with TGFβ1 and suppresses p38 and smad3 phosphorylation with the consequence of decreasing extracellular vesicle release by VSMCs and ALP contained within.

In a more recent study, Cerrado et al. (96) have revealed that calcific aortic valve stenosis is increased in CML patients who receive nilotinib (a TKI) treatment. Surprisingly, nilotinib targets DDR2 in human VICs, suggesting that DDR2 plays a crucial role in heart valve calcification. A clinical study showed the decrease of DDR2 expression in CKD patients in which valvular diseases are highly prevalent. Therefore, these studies implicate that DDRs, either DDR1 or DDR2, have an important role in cardiovascular calcification.

### C-MET

Hepatocyte growth factor (HGF) binds to its specific receptor, c-Met. C-Met is a member of TK in the HGFR family. The involvement of HGF in cardiovascular diseases has been reviewed elsewhere (97). Recent findings have suggested the role of c-Met in inducing VC. Liu et al. (98) have demonstrated the upregulation of c-Met during VSMC osteogenic differentiation. Through HGF overexpression, the c-Met/Akt/Notch3 signaling was increased, and osteogenic markers, Runx2, osteocalcin, BMP2, and osterix, were also increased. Intriguingly, another group has observed similar results. Inducing VSMC calcification using β-glycerophosphate increased c-Met at day 21. Surprisingly, treatment with a c-met inhibitor, crizotinib (a TKI), has led to VC amelioration (99).

### Tie receptor

Tie2 is well known due to its signature in endothelial cells, as many studies on specific gene knockout in endothelial/endocardial cells target the Tie2 promoter (100). Angiopoietin-2 (angpt2) is the ligand of another tyrosine kinase family, Tie. Treatment with angpt-2 has enhanced the calcification of the arterial ring in CKD patients. A Tie-2 receptor has also been detected. This finding suggests that the angpt-2-Tie-2 signaling may play a role in VC (101). Moreover, Jeong et al. have confirmed that the Tie-2 receptor and its ligand, Ang1, were expressed in differentiating osteoblasts at day 3 and maintained up to 25 days (102).

Arevalos et al. (103), in an effort to link neovascularization with CAVD, have reported that the angiopoetin1-Tie-2 signaling contributes to the calcified aortic valve disease (CAVD) progression. VICs have been demonstrated to have pericyte-like behavior and undergo angiogenic-like phenotype depending on the angiopoetin1-Tie-2 signaling. This evidence proves the concept of neovascularization, in which Ang1-Tie-2 is the common pathway, and CAVD by the VIC/VEC interaction and Ang1-Tie2 dynamics.

### ROR and RYK

ROR and RYK families of RTK were initially considered orphan receptors due to the lack of information regarding their ligands. However, recent findings have shown that ROR and RYK families have a functional extracellular Wnt-binding domain and can transmit Wnt-signal transduction (104). ROR and Ryk are involved in cardiovascular disease and are thought to activate non-canonical WNT pathways signaling; thus, they have been thoroughly reviewed (105). In the context of cardiovascular calcification, the Wnt5a-Ror2 pathway has been reported to regulate VC (106). Indeed, in an effort to inhibit VC by using STAT3 siRNA, ROR2 and RUNX2 expression was strongly decreased (107), confirming the role of ROR2 in inducing VC. Moreover, the evidence of ROR2 interaction with MSX2, one of the transcription factors involved in osteoblast-like cell differentiation (108), has been demonstrated (109).

## Evidence linking cancer, tyrosine kinase inhibitors, and cardiovascular calcification

Most cancers are associated with the aberrant activation of TK signaling (2). The evidence linking cancer and VC has come from two independent studies: the Multi-Ethnic Study of Atherosclerosis (MESA) study (110) and the CAC consortium (111). In the MESA study, Whitlock et al. have shown that the incidence of coronary artery calcification (CAC) was independently associated with cancer history. The diagnosis of cancer was associated with the development of CAC even after accounting for atherosclerotic risk factors (110). Another study assessed the relationship of CAC with CVD and cancer mortality and found a relationship between CAC with CVD and cancer mortality, which significantly differed for women and men (111). Additionally, a molecular study has shown an increase in CAV-1 in calcifying VSMC (27), which is similar to malignancy (112).

The widespread use of TKIs in cancer and the evidence of TKI-associated cardiotoxicity are increasing and have been reviewed elsewhere (113). Imatinib, a TKI used to treat CML, has been reported to induce bone remodeling dysregulation as it targets cells of the skeleton and affects osteoblasts and osteoclasts (114). Additionally, elevated risk for cardiovascular complications, such as hypertension, heart failure (HF), left ventricular systolic dysfunction, myocardial ischemia (MI), and QT interval prolongation, has been reported in cancer patients receiving TKI treatment (4, 5). It seems that TKI-related cardiotoxicities have preferential occurrence in patients with preexisting comorbidities (115). Hurley et al. (116) reported that patients who have a history of hyperlipidemia, congestive heart failure, coronary artery diseases, and chronic obstructive pulmonary disease before TKI treatment have a greater risk of developing TKI-related cardiotoxicity. Interestingly, data reanalysis by Moslehi and Deniger (117) revealed important information regarding risk factors underlying the cardiovascular adverse effect of TKI. Patients who have traditional risk factors of atherosclerosis, such as hypertension, diabetes, and age, are predisposed to serious adverse effects. The combination of two or more traditional risk factors even doubles the risk of developing more severe cardiovascular toxicity. These data have great implications in the practice before starting treatment with TKI and deciding on personalized treatment. In addition to preexisting comorbidity, some TKIs have unique adverse event profiles (115) (see also Table 1). Nilotinib has the greatest proportion of cardiovascular adverse effects (Table 1). The data analyzed by others showed similar results (118, 119). Additionally, clinical and molecular studies support the role of nilotinib in promoting aortic valve calcification (43, 96). Consequently, the experts recommend avoiding nilotinib in patients having cardiovascular disease history (119, 120). Current experts' opinions regarding the safest TKIs in relation to cardiovascular events are imatinib and bosutinib (120). Moreover, molecular studies comparing first-generation and second-generation TKI, imatinib and nilotinib, in cardiovascular calcification further supports this recommendation (43).

As the TKs are distributed in cardiovascular tissues, the cardiotoxicity of TKI cannot be fully eradicated despite of “safer” TKI profile in the cardiovascular system. Therefore, specific drug delivery is required. Recently, an effort to address this issue has been made. The current advances in drug delivery give some hope to this issue. The use of nanomedicine has been applied to precisely deliver anticancer drugs to specific cancer cells, avoiding the extrinsic off-target effect of antineoplastic agents (121). Nanoparticles can achieve the cancer tissue through passive and active targeting and triggered release. Passive targeting can be achieved by enhanced permeability and retention (EPR) effects, whereas active targeting can be achieved by modifying the nanoparticle surface with specific desired peptides (122). Interestingly, nanomedicine has been used to treat cardiovascular diseases, such as atherosclerosis (123, 124). Therefore, nanomedicine could be the future tool for precision medicine for addressing TKI-associated cardiotoxicity.

## Perspective and conclusion

We found 11 RTKs that are involved in the regulation of cardiovascular calcification. These RTKs and their role in cardiovascular calcification are summarized in Table 2. RTKs play a crucial role in cardiovascular calcification. They possess two facets in cardiovascular calcification, which can be either stimulatory or inhibitory effect, depending on the ligand and tissue. Therefore, inhibiting RTKs could theoretically benefit and/or disadvantage cardiovascular calcification. Despite the vast evidence on TKI-induced cardiotoxicity, TKIs can be beneficial to the cardiovascular system. For instance, targeting EGFR using TKI (erlotinib) improves atherosclerosis in mice (17). Moreover, a selective TKI for EGFR (ErB1) has been demonstrated to alleviate VC (27). Additionally, crizotinib can ameliorate VC as well (99). Interestingly, gefitinib, a TKI targeting EGFR, has the safest profile in cardiovascular adverse effects (Table 1). The combination of TKI with cardiovascular protectant agents such as statin could also be a beneficial strategy in combating cancer and TKI-related cardiotoxicity. The synergy between TKI and statin in cancer cell killing has been demonstrated in clinical and molecular studies (125–128). Considering the evidence of cancer-associated VC, TKI-benefiting vascular system, and the synergy between TKI and statin in cancer cytotoxicity, the EGFR-related cancers with the atherosclerotic disease might have the most benefit from the treatment with TKI and statin combination. However, this requires further confirmation.

**Table 2:** Summary of RTKs involvement in cardiovascular calcification.

**RTK**	**Vessel calcification**			**Ref**.	**Heart's Valve calcification**			**Ref**.	**Other tissues**			**Ref**.
	**Experimental design**	**Signaling pathway target**	**Conclusion**		**Experimental design**	**Signaling pathway target**			**Experimental design**	**Signaling pathway target**	**Conclusion**	
EGFR	Clinical study SNP association; CKD-induced calcification mice; hVSMC	CAV1-associated vesicle; TNAP	Induce vessel calcification	(27)	*Egfr* conditional KO mice	Epithelial-to-mesenchymal transition or osteogenic activity marker	Suppress valve calcification	(31)			–	–
PDGFR	*PGDGFBB* overexpressed TG mice; *PDGFBB* KO mice; HFD mice induced arterial stiffening	PDGFRβ; Osteogenic transdifferentiation through RUNX2	Induce vessel calcification	(38)			–		Site directed mutagenesis of *PDGFRB*; pharmacological stimulation of with PDGF and inhibition of Akt in mesenchymal cell isolated from human bone marrow aspirate	Akt-MAPK;	↓ Brain VC ↓ bone matrix mineralization	(40–42)
FGFR-FGF2	Human atherosclerotic artery; bovine VSMC and human VSMC line; FGFR inhibitor and *Syndecan4* KD	Akt-Erk; TGFβR-PKCα	Induce vessel calcification	(46)	Human heart leaflets; FGFR pharmacological inhibition of VIC isolated from porcine	Akt-mTOR	Suppress valve calcification	(52)			–	–
FGFR-FGF21	Vitamin D3 + nicotine induced calcification rat; pharmacological stimulation with FGF21	Endoplasmic reticulum stress induced apoptosis pathway	Suppress vessel calcification	(48)			–	–			–	–
FGFR-FGF23	Nephrectomy induced CKD mice; FGFR pharmacological inhibition and FGF23 pharmacological stimulation	ERK pathway	Induce vessel calcification	(50)	Pharmacological stimulation with FGF23 and pharmacological inhibition with FGFR inhibitor in human derived AVIC	N/A	Induce valve calcification	(58)			–	–
IGFR	Pharmacological stimulation with IGF and glycosylation inhibitor in VSMC line	Akt-MAPK	Suppress vessel calcification	(63, 64)	Human VIC derived from aortic valve replacement surgery and VIC derived from e*NOS* KO mice	eNOS KO → Upregulation DPP4 enzyme → IGF1 breakdown → IGF1-IGFR binding reduction →	Inhibit valve calcification	(70)			–	–
VEGFR			–	–	Pharmacological stimulation with VEGF, inhibition with CaMKII inhibitor, and IP3R inhibitor in porcine-derived VIC	IP3R/CaMKII/CREB/ Runx2 pathway	Induce valve calcification	(73)	*VEGF*^120/120^ KO mice	*VEGF*^120/120^ KO mice reduced osteoblastic activity	↑ Bone growth	(74)
AXL	Overexpression of Axl in VSMC	PI3K/Akt	Suppress vessel calcification	(76–79, 83)			–	–			–	–
TRK	Pharmacological stimulatiof of TrkB of ostegeogenic medium induced HUVEC calcification	Activation of TrkB suppress VC by inhibit EndMT through TrkB/KLF2 pathway	Suppress vessel calcification	(90)	Overexpression of NT3 (ligand of Trk) and pharmacological stimulation	Trk-Akt pathway	Induce valve calcification	(89)	Pharmacological stimulation and inhibition of TrkA in human articular chondrocyte	IHH-PTHrP signaling	↑ Chondrocyte matrix mineralization	(87)
DDR	DDR1/LDLR single and double KO mice	PI3K/Akt/Runx2 pathway	Induce vessel calcification (DDR1)	(92, 93)	TKI treated VIC derived from Hyperlipidemic APOE*3Leiden.CETP transgenic mice; gene expression analysis of human derived heart valve undergoing valve surgery	N/A	Suggest the suppression of heart valve calcification through DDR2	(96)			–	–
c-MET	Overexpression of cMET ligand (HGF) in VSMC line; Overexpression of HGF antagonist	Promote osteogenic differentiation c-Met/Akt/Notch3 signaling	Induce vessel calcification	(98, 99)			–	–			–	–
TIE2	Pharmacologic stimulation with Tie2 ligand in smooth muscle cell derived from children undergoing renal transplantation	N/A	Induce vessel calcification	(101, 102)	Pharmacological inhibition of Tie2 in VIC and VEC derived from porcine heart valve	Akt pathway	Induce valve calcification	(103)			–	–
ROR2	Osteogenic induction in hADSC; pharmacological stimulation and STAT3 knock down	STAT3 dependent ROR2-RUNX2 signaling	Induce vessel calcification	(107)			–	–			–	–

↑ means increased.

↓ means decreased.

A growing body of epidemiological evidence suggests the association of malignancy with VC. Molecularly, cancer-associated RTK, depending on the ligand and tissue, has varying effects on cardiovascular calcification (vessel and heart valve calcifications) and can induce or inhibit cardiovascular calcification. Currently, a few clinical data regarding TKIs and cardiovascular calcification exist, warranting further research.

## Author contributions

AM and P-YL: conceptualization. AM: methodology, formal analysis, and investigation. AM, W, MR, and P-YL: writing—original draft preparation and writing—review and editing. AM: visualization. W, MR, and P-YL: supervision. AM, W, and P-YL: project administration. P-YL: funding acquisition. All authors have read and agreed to the published version of the manuscript.

## Funding

This study was supported by grants 108-2314-B-006-098-MY3, 109-2314-B-006-068-MY2, and 111-2314-B-006-017-MY3 from the Ministry of Science and Technology of Taiwan, and the grant of D111-G2512 from Higher Education Sprout Project, Ministry of Education to the Headquarters of University Advancement at National Cheng Kung University.

## Conflict of interest

The authors declare that the research was conducted in the absence of any commercial or financial relationships that could be construed as a potential conflict of interest.

## Publisher's note

All claims expressed in this article are solely those of the authors and do not necessarily represent those of their affiliated organizations, or those of the publisher, the editors and the reviewers. Any product that may be evaluated in this article, or claim that may be made by its manufacturer, is not guaranteed or endorsed by the publisher.
